# Detection of cytokine release syndrome using wearables and cytokine profiling following CAR-T therapy for myeloma

**DOI:** 10.1172/jci.insight.203988

**Published:** 2026-05-05

**Authors:** Sridevi Rajeeve, Matt Wilkes, Nicole Zahradka, Lewis Tomalin, Mujahid Quidwai, Darren Pan, Nicholas J. Calafat, Martin Cusack, Adolfo Aleman, Kseniya Serebryakova, Katerina Kappes, Hayley Jackson, Sarita Agte, Santiago Thibaud, Larysa Sanchez, Shambavi Richard, Joshua Richter, Cesar Rodriguez, Hearn Jay Cho, Ajai Chari, Sundar Jagannath, Alessandro Laganà, Adriana C. Rossi, Samir Parekh

**Affiliations:** 1 Myeloma & Cellular Therapy Services, Memorial Sloan Kettering Cancer Center, New York, New York, USA.; 2 Current Health Inc., Boston, Massachusetts, USA.; 3 Department of Population Health Science and Policy and; 4 Department of Oncological Sciences, Icahn School of Medicine at Mount Sinai, New York, New York, USA.; 5 University of California San Francisco, San Francisco, California, USA.; 6 Department of Medicine, Hematology and Medical Oncology and; 7 Tisch Cancer Institute, Icahn School of Medicine at Mount Sinai, New York, New York, USA.; 8 Dorset County Hospital, NHS Foundation Trust, Dorchester, United Kingdom.; 9 Department of Genetics and Genomic Sciences, Icahn School of Medicine at Mount Sinai, New York, New York, USA.

**Keywords:** Hematology, Immunology, Oncology, Biomarkers, Cancer immunotherapy, Clinical practice

## Abstract

**BACKGROUND:**

Chimeric antigen receptor T-cell (CAR-T) therapies have revolutionized treatment for relapsed/refractory multiple myeloma (RRMM). However, cytokine release syndrome (CRS), a common and potentially severe complication, requires inpatient monitoring, limiting access and increasing costs. Wearable devices could support outpatient CAR-T delivery, but feasibility for CRS detection versus standard care remains unproven.

**METHODS:**

We conducted a prospective, single-center observational pilot study to assess the feasibility of using wearable devices for monitoring vital signs and detecting CRS. Thirty patients receiving idecabtagene vicleucel (ide-cel) or ciltacabtagene autoleucel (cilta-cel) were enrolled; 25 with sufficient monitoring data were evaluable. Sensors collected skin and axillary temperature, oxygen saturation, respiratory and heart rate, and motion. Peripheral blood cytokines were analyzed pre- and postinfusion using a multiplex proteomic platform. The primary outcome was feasibility, assessed by CRS detection sensitivity and specificity; secondary outcomes included adherence, lead time, and performance of models integrating wearable and cytokine data.

**RESULTS:**

CRS occurred in 20 of 25 patients. The best-performing wearable model detected 18 or 20 CRS episodes with a sensitivity of 0.72 (mean 0.75; 95% CI 0.60–0.91) and a specificity of 0.80 (mean 0.76; 95% CI 0.68–0.84), and a median lead time of 7:00 hours before nursing recognition. Median adherence during high-risk periods was 71%. Cytokine changes paralleled temperature elevations, and IFN-γ emerged as a consistent biomarker.

**CONCLUSION:**

Wearable devices are feasible for early CRS detection and may support outpatient CAR-T care. Larger outpatient studies are warranted.

**TRIAL REGISTRATION:**

This study did not meet the criteria for ClinicalTrials.gov registration.

## Abstract 2

Wearable temperature sensors detected early signs of CAR-T treatment toxicity in myeloma patients hours before standard hospital monitoring, supporting safer outpatient care.

## Graphical abstract

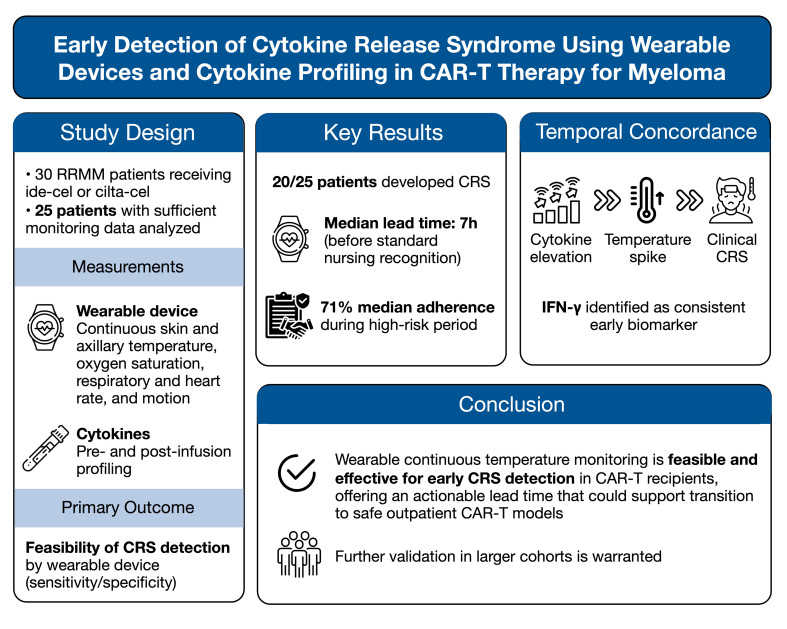

## Introduction

The advent of chimeric antigen receptor T cell (CAR-T) therapies has transformed treatment for relapsed/refractory multiple myeloma (RRMM). CAR-T therapies engineer patients’ T cells to express receptors (CARs) targeting specific cancer cells, enhancing immune responses. Two FDA-approved CAR-T therapies targeting B cell maturation antigen (BCMA), idecabtagene vicleucel (ide-cel) (1) and ciltacabtagene autoleucel (cilta-cel) (2), have yielded unprecedented responses. Despite this success, barriers to widespread use include limited apheresis (blood cell collection) slots, lengthy manufacturing processes, and the need for proximity to specialized centers for toxicity monitoring and caregiver support (3).

Currently, most institutions adopt an inpatient CAR-T therapy model, including conditioning chemotherapy, CAR-T infusion, and postinfusion monitoring for side effects like cytokine release syndrome (CRS) and immune effector cell–associated neurotoxicity syndrome (ICANS) over 10–14 days (4). However, prolonged hospital stays may increase the risk of iatrogenic infections, while increasing financial strain and resource utilization. The FDA has recently removed the Risk Evaluation and Mitigation Strategy (REMS) requirements around CAR-T, and these drugs are also expected to receive approval in earlier treatment lines. These changes will drive expansion in use but also make safe and efficient delivery essential, particularly in community oncology settings.

CRS is a key immunologic adverse effect of CAR-T and bispecific antibodies (bsAbs), defined by the American Society of Transplantation and Cellular Therapy (ASTCT) as a supraphysiologic immune response triggered by T cell activation (5). CRS onset typically occurs 1–2 days after ide-cel infusion and 6–9 days after cilta-cel infusion, with most cases being mild to moderate (Grade 1–2) (1, 2, 6). These timelines support hybrid inpatient/outpatient protocols, provided that appropriate toxicity-management resources are in place (7).

Continuous, remote monitoring of vital signs is now feasible through wearable device technology (8–11). Initially used in cardiology (12), wearables gained prominence during the COVID-19 pandemic and are now applied in oncology settings, including hospital-at-home, cellular therapy programs, and exploratory outpatient care. Adoption has been driven by necessity but there remain few direct comparisons to inpatient care (13). Wearable devices could enable rigorous monitoring for CRS while reducing hospital stays (10, 14, 15). Correlating these biomarkers with wearable data and outcomes could help identify high-risk patients early, a critical step for outpatient management. Tracking serological biomarkers of T cell activation and CRS offers additional insights into CRS mechanisms and toxicity reduction strategies (16).

This investigator-initiated trial used wearable devices for continuous monitoring of vital signs in patients with RRMM undergoing CAR-T therapy, along with cytokine monitoring to track CRS dynamics. We aimed to assess feasibility and develop proof-of-concept models for early CRS prediction. We conducted this trial in an inpatient setting to offer a within-subject comparison with standard care as a foundation for future studies with simpler wearable devices and serological biomarkers in outpatient settings.

## Results

**Figure 1:**
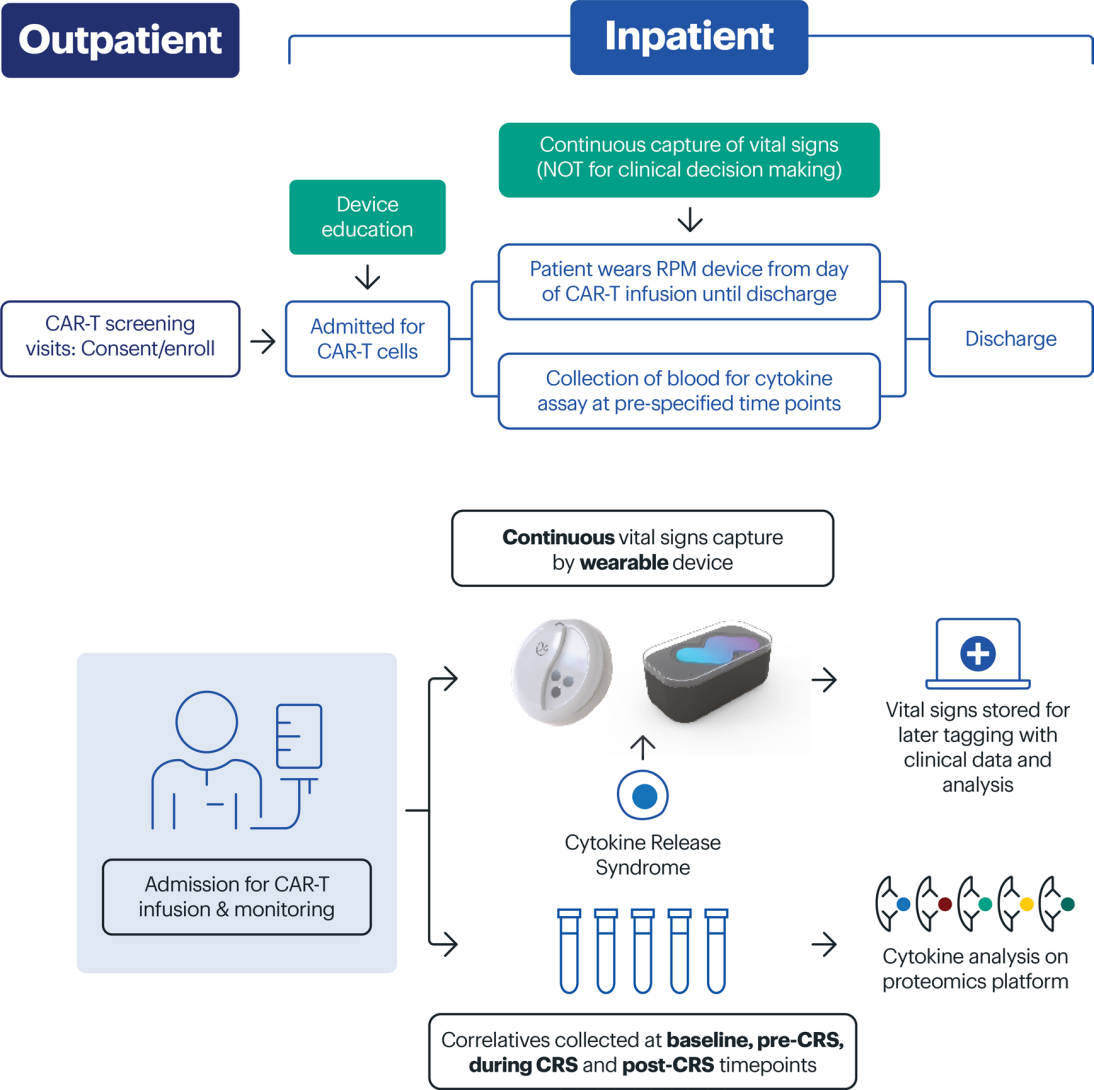
Study schema.

Thirty patients were enrolled in the trial. The median (IQR) length of stay was 13 (12 to 14) days. One patient was excluded from the CRS detection analysis due to concurrent SARS-CoV-2 infection. Of the remaining 29 patients, 25 developed CRS, predominantly Grade 1 (*n* = 22), with fewer patients experiencing Grade 2 (*n* = 1), Grade 3 (*n* = 1), and Grade 4 (*n* = 1) events (see Figure 1, Table 1, Supplemental Table 1, and CONSORT diagram in Supplemental Figure 1; supplemental material available online with this article; https://doi.org/10.1172/jci.insight.203988DS1).

### Wearable temperature monitoring enables early CRS detection with high specificity and feasibility in patients who were hospitalized.

Wearable adherence, defined as the proportion of valid sensor observations (nonnull readings) to the total number of possible observations based on the sensor’s sampling rate (every 2 seconds for skin temperature, pulse rate, SpO2, and motion; every 4 seconds for respiratory rate), was 67% (IQR: 52%–78%) overall and 71% (IQR: 55%–84%) during product-specific high-risk periods (days 0–5 postinfusion for ide-cel; days 5–11 for cilta-cel). This was achieved in a hospital setting with blinding to device outputs and utility. Temperature analysis included 25 of 29 patients, excluding 4 due to insufficient axillary temperature data prior to CRS episodes. Most gaps in wearable data occurred when the patients left the floor or the device was removed briefly for personal care.

To optimize the detection of CRS, we tested various observation windows (time for aggregating vital signs) and step sizes (overlap between windows), finding a 60-minute window with a 10-minute step size feasible. Longer windows can smoothen out transient fluctuations, while larger steps reduce data processing (Supplemental Figure 2). These settings can guide home-use applications where daily activities introduce variability (17).

The best model combined axillary temperature with individual baselines and a fixed 36.4°C threshold (observation window: 60 minutes, step size: 10 minutes), detecting 18 of 20 CRS episodes with a sensitivity of 0.72 (mean 0.75; 95% CI 0.60–0.91), specificity of 0.80 (mean 0.76; 95% CI 0.68–0.84), and a median lead time of 7:00 hours (IQR: 3:16) before standard nursing recognition. A simpler fixed threshold of 38.0 °C, corresponding with the ASTCT definition of grade 1 CRS detected only 12 episodes, with a median lead time of 1:45 hours (Table 2 and Supplemental Table 2).

### Distinct cytokine profiles and temporal patterns differentiate CRS onset across CAR-T products.

Currently, CRS in patients with RRMM is managed per ASTCT guidelines, developed primarily for CD19-directed CAR-T cells. While clinical manifestations are broadly similar, CRS onset and severity vary across CAR-T products, suggesting distinct cytokine responses.

**Figure 2:**
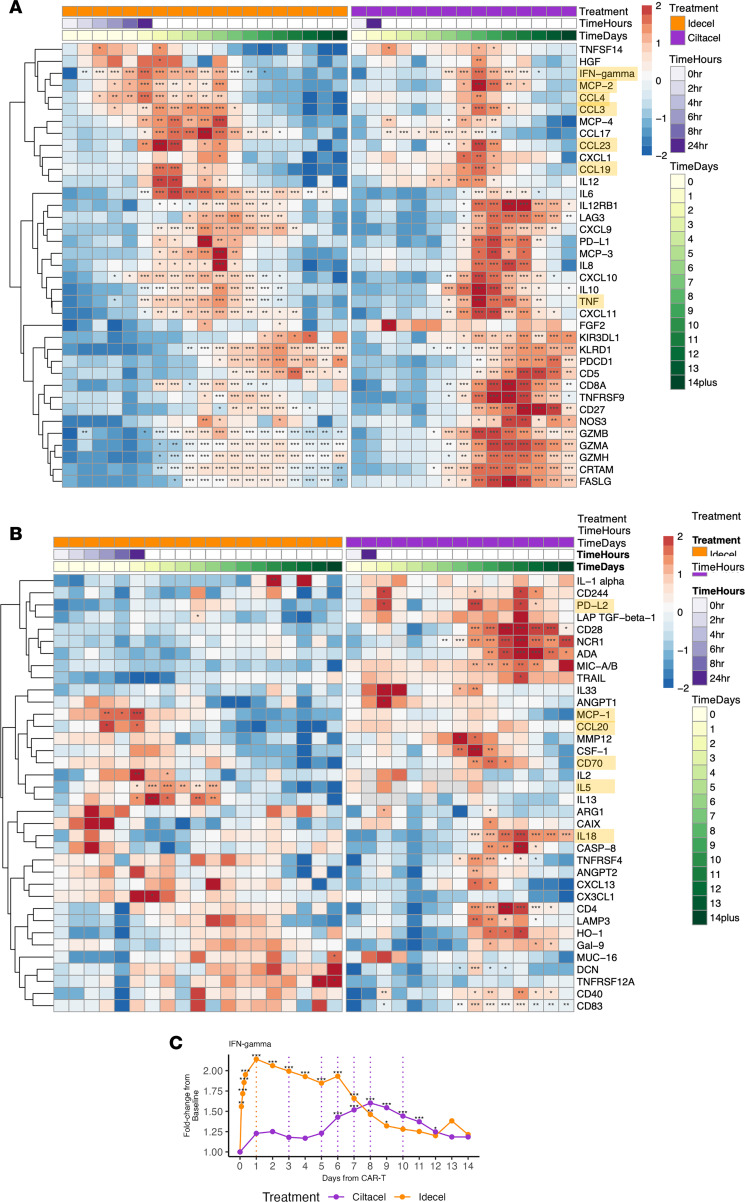
Olink analysis reveals distinct timing of peak inflammation following ide-cel and cilta-cel. (**A** and **B**) The heatmaps show scaled model-estimated mean expression of inflammatory biomarkers following treatment with cilta-cel or ide-cel. Biomarkers showing significant changes in both treatment groups are shown in **A**, whereas biomarkers showing treatment-specific changes are shown in **B**. Longitudinal expression of each inflammatory marker was analyzed using a linear mixed-effects regression model fit with the DREAM framework, with time as a fixed effect, age as a covariate, and random intercepts for patient ID and plate ID. Comparisons of interest were performed using contrasts, and *P* values were adjusted for multiple testing using the Benjamini-Hochberg method. Asterisks indicate significant change from baseline at the indicated time point (fold-change > 1.3). (**C**) Model-estimated fold-change from baseline for IFN-γ following ide-cel or cilta-cel treatment. Fold-changes and significance were derived from the same linear mixed-effects regression/DREAM analysis described above, with Benjamini-Hochberg correction for multiple testing. Asterisks indicate significant change from baseline at each time point (* *FDR* < 0.05, ** FDR < 0.01, *** FDR < 0.001).

As an exploratory analysis complementing the primary wearable feasibility assessment, we collected peripheral blood samples at predetermined intervals and analyzed them using the Olink Proteomics Platform (see Methods). Longitudinal inflammatory profiles revealed 73 inflammatory markers with significant changes following CAR-T treatment, clustering into distinct temporal patterns (Figure 2, A and B). While most cytokines changed in both treatments, some were treatment-specific changes. Twenty-six cytokines significantly changed specifically following cilta-cel, including IL-18, CD28, and CD70, while ide-cel–specific increases included 10 cytokines such as CCL20, ARG1, and IL-1α, although these were transient. MCP-1 and IL-5 showed more sustained increases with ide-cel than cilta-cel (Supplemental Figure 3).

IFN-γ, an early inflammatory marker, rose sharply within 24 hours after ide-cel infusion, with a fold-change of 2.1 (FDR < 0.001), and peaked earlier than cilta-cel, where levels increased more gradually and peaked at 1.6 fold-change by day 8 (Figure 2C). This timing mirrored CRS onset, which was delayed with cilta-cel. Similar treatment-specific temporal patterns were observed for MCP-2, TNF, CCL3, CCL23, CCL4, and CCL19, although IFN-γ showed the largest fold-change from baseline to peak (Figure 2C).

Considering the prominent early increase in IFN-γ and the central role of fever in CRS, we next examined whether IFN-γ dynamics were associated with physiologic temperature measurements captured by the wearable devices. To estimate the within-patient association between axillary temperature and IFN-γ while accounting for repeated observations from the same individual, we employed repeated-measures Pearson correlation implemented in the *rmcorr* R package. Using this approach, we observed a moderate positive association between mean daily temperature and daily IFN-γ NPX score (r = 0.50, *P* < 0.001), suggesting that IFN-γ–driven inflammatory activity may contribute to the physiologic temperature elevations detected by wearable monitoring during CRS development (Supplemental Figure 4).

### IFN-γ fold-change as a predictive biomarker for CRS.

**Figure 3:**
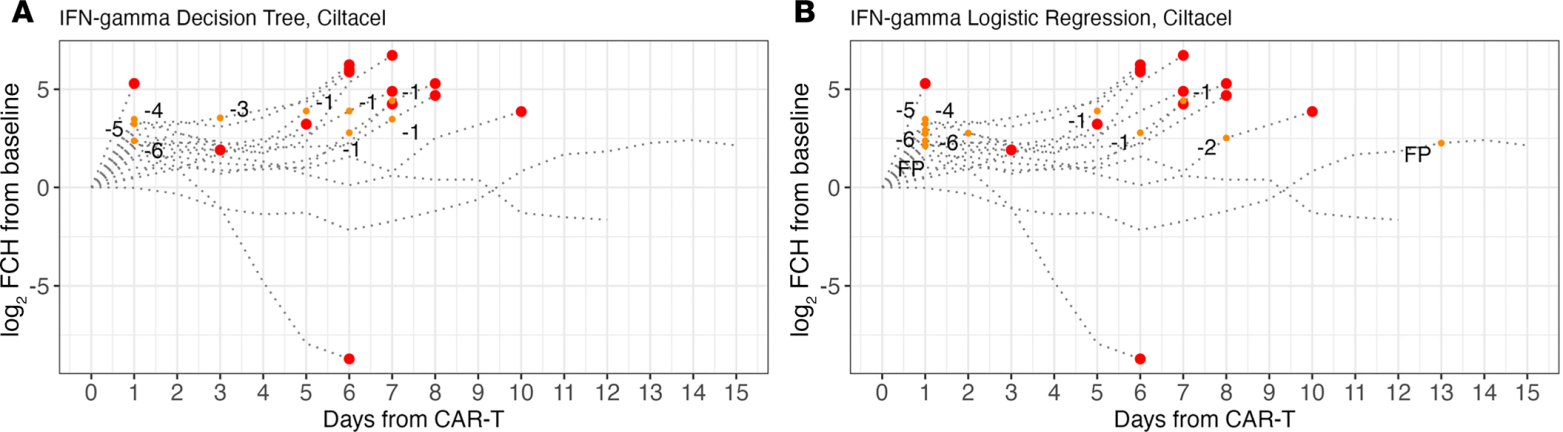
IFN-γ fold-change as a predictive biomarker for CRS. (**A**) Decision tree model (**B**) and logistic regression model using IFN-γ log_2_ fold-change from baseline in patients treated with cilta-cel. Points represent individual observations, and dashed curves illustrate the fitted decision boundaries of the model across time from CAR-T infusion. The red points indicate the day that CRS occurred for each patient, orange labels indicate lead time for each patient, ‘FP’ indicates False Positive (i.e. patient never experienced CRS during the study).

Given the substantial changes in IFN-γ following CAR-T therapy, we evaluated its potential as a simple, single-biomarker predictive marker for CRS onset that could be paired with wearable monitoring in future outpatient protocols. Using daily IFN-γ fold-change from baseline, we developed a decision-tree classifier for cilta-cel patients, achieving a precision of 100%, sensitivity of 75%, specificity of 100%, accuracy of 80%, and F1 score of 85.7%, correctly identifying 9 of 12 CRS patients with no false positives (Figure 3). Patients were identified with a median lead-time of 24 hours before CRS onset (min = 24, max = 144). A logistic regression model performed similarly (precision = 83.3%, sensitivity = 83.3%, specificity = 33.3%, accuracy = 73.3%, F1 = 83.3%) (Table 3).

For ide-cel, the decision-tree classifier achieved 83.3% precision, 62.5% sensitivity, 0% specificity, 55.6% accuracy, and F1 of 71.4%, while logistic regression had similar performance (precision = 80%, sensitivity = 50%, specificity = 0%, accuracy = 44.4%, F1 = 61.5%) (Supplemental Figure 5). Low specificity reflected the limited sample size, as only one non-CRS patient was included in the ide-cel cohort. While the performance of these classifiers is likely not generalizable due to the small sample size, the high precision suggested potential predictive value for IFN-γ, warranting validation in larger cohorts.

### Exploratory integration of temperature and cytokine data.

In further exploratory analysis, we asked whether combining temperature with cytokine levels could suggest improved CRS prediction. For each product (Ide-cel and Cilta-cel), two models were developed: (a) cytokines only and (b) cytokines plus wearable data, for a total of four models. Among 5 classifiers tested (see Methods), Gradient Boosting was optimal for ide-cel (F1: 81%, accuracy: 77%, precision: 86%, sensitivity: 77%, specificity: 67%), and Random Forest for cilta-cel (F1: 82%, accuracy: 79%, precision: 86%, sensitivity: 78%, specificity: 70%). For context, a naive majority-class baseline that always predicts no-CRS (the majority class comprising 82% of ide-cel and 81% of cilta-cel observation) would achieve comparable accuracy but 0% sensitivity and 0% F1 for CRS detection, demonstrating that our models learn a clinically meaningful decision boundary (see Supplemental Table 3). Unlike fixed temperature thresholds used clinically to trigger CRS evaluation (Table 2), these models do not rely on a single physiologic cutoff; instead, they learn multivariable patterns associated with CRS from longitudinal biomarker data.

Key predictive biomarkers varied by product, with IL-5, IFN-γ, NOS3, CD4, and TNFRSF9 most important for ide-cel, and IL-10, CCL4, MCP-2, MCP-3, and IFN-γ for cilta-cel (Supplemental Figure 6). IFN-γ’s presence in both models highlighted its potential role in CRS detection.

Our analysis above demonstrated that both axillary and skin temperature monitoring using wearable devices accurately detected CRS ahead of standard nursing care. Therefore, we hypothesized that adding wearable temperature data would enhance model performance. Skin temperature measured from the upper arm wearable was chosen over axillary due to continuous collection, better adherence, and ease of future deployment. While skin temperature was affected by ambient conditions and skin perfusion, it closely tracked axillary temperature (typically 1–2°C lower) during febrile events. We applied interpolation to align the less frequent cytokine measurements with continuous temperature data for model input (see Methods). Combined models integrating temperature and cytokine data showed mixed results across evaluation metrics: while overall accuracy increased (ide-cel: 85%, Random Forest; cilta-cel: 81%, Gradient Boosting), precision and sensitivity declined relative to cytokine-only models (ide-cel: precision 67%, sensitivity 50%, F1 57%; cilta-cel: precision 43%, sensitivity 38%, F1 40%), though specificity improved for cilta-cel (88%) within a 6-hour prediction window (Supplemental Figure 7). These trade-offs suggest that adding skin temperature may help reduce false positives but does not uniformly enhance prediction, likely reflecting the challenge of aligning interpolated cytokine data with continuous temperature measurements. Time-lagged features, defined as the rate of change between consecutive measurements at 6-, 12-, and 24- hour intervals (ΔC = C(t) − C(t−lag)), were included to capture dynamic biomarker trajectories. While these features contributed to the models, their independent effect on performance could not be reliably isolated, given the small sample size. Overall, these results suggest that integrating physiologic temperature trajectories with cytokine signals may improve discrimination of CRS compared with simple fever thresholds, although larger datasets will be required to fully realize the predictive potential of this multimodal approach. Although underpowered, these exploratory findings are encouraging and provide a rationale for future studies evaluating multimodal biomarker models for earlier CRS detection. Complete performance metrics for CRS classification models are provided in Supplemental Table 3.

## Discussion

Indications for CAR-T therapy are expanding across MM and other malignancies. However, geographical limitations and provider confidence typically restrict access to tertiary centers. Decentralizing CAR-T therapy with outpatient monitoring could improve accessibility, allowing patients to benefit from the removal of the REMS restrictions. In some US trials, outpatient CAR-T models using preemptive admission for tocilizumab and dexamethasone have reduced overall admissions but not fully mitigated prolonged or severe CRS episodes (18, 19). Wearable devices, particularly those measuring temperature continuously, support outpatient and community models by enabling actionable monitoring and reducing prolonged hospital stays, with their attendant risks of infection and immobility. (20–22)

The primary outcome of this study was the feasibility of detecting the onset of CRS with a wearable device in CAR-T therapy patients, definitively validating the approach in an inpatient setting. Detection of 18 of 20 CRS episodes using axillary temperature from the wearable resulted in a median lead time of 7:00 hours before standard nursing care. Both axillary and skin temperature emerged as reliable biomarkers, offering immediate utility for early CRS detection and intervention. Patient adherence was 71% (range 55%–84%) during high-risk periods. These findings should build clinician confidence that wearable devices can enable early detection and intervention for CRS. We believe that the greater forewarning of CRS offered by these devices will be especially reassuring to clinicians seeking to expand services further away from treatment centers. Challenges like user error and adherence during critical times remain (17). Adherence in home settings, with regular check-ins, may exceed those recorded in this ‘black box’ inpatient study, as the utility of the wearables will be more apparent to patients and caregivers (22, 23). We note that wearables are only one part of an outpatient program, which also requires robust home-based care coordination to respond to alerts and offer prompt treatment (24).

In our exploratory work, the study highlighted the importance of peripheral blood cytokine assessments for CRS monitoring, particularly in inpatient settings where they play a crucial role in guiding treatment decisions (25). We observed postinfusion shifts in cytokine levels, which captured the immune response associated with CAR-T–induced CRS. Key cytokines like IFN-γ, TNF, and chemokines (e.g. MCP-2, CCL3, CCL23) showed distinct profiles between ide-cel and cilta-cel. IFN-γ, a central mediator in T cell activation and macrophage stimulation, demonstrated reliable precision in predicting CRS and emerged as a reproducible and complementary biomarker, supporting integration with temperature monitoring for early prediction of impending CRS. Notably, the multicytokine ML models, which incorporated the full panel of cytokines, identified product-specific signatures (e.g., IL5, NOS3, and CD4 for ide-cel; IL10, CCL4, and MCP-2 for cilta-cel) and achieved higher F1 scores than the single-biomarker IFN-γ classifier. However, IFN-γ was highlighted as a practical single-biomarker candidate because of its consistency across both products and the feasibility of rapid turnaround assays (e.g., ELLA), balancing predictive value with clinical deployability. IFN-γ displayed the earliest and most pronounced increase following ide-cel, consistent with its role in driving cytokine release and its known association with CRS onset timing in CAR-T therapies (26, 27). A delayed IFN-γ peak in cilta-cel correlated with later CRS onset, reflecting product-specific immune activation patterns.

Recent studies reinforce the potential of cytokines like IFN-γ, IL-6, and IL-10 as biomarkers for predicting CRS severity, highlighting an additional use beyond their utility in early CRS detection (28). Our findings of distinct cytokines, including IL-18, CD28, and CD70 uniquely elevated with cilta-cel, and IL-1α, ARG1, and MCP-1 transiently elevated with ide-cel, support the idea of individualized cytokine signatures that could aid in predicting CRS risk and severity based on CAR-T product type. MCP-1, a chemokine induced by IL-1 signaling, may serve as an indirect readout of IL-1 activity, highlighting its potential role in inflammatory pathways driving CRS (29, 30). These results underscored the need for personalized cytokine monitoring in CRS management, aligning with emerging evidence that targeted cytokine panels may help stratify patients based on their risk for severe CRS and guide tailored intervention strategies.

Ultimately, the goal would be to combine wearable vital sign and cytokine monitoring in a readily deployable, scalable outpatient pathway. For cilta-cel, a practical model could involve discharging patients after infusion with the wearable device in place and returning them to clinic daily for a rapid multiplex cytokine panel (e.g., ELLA), the approximately 2-hour turnaround of which could allow same-day intervention if temperature or IFN-γ thresholds signal impending CRS. For ide-cel, where median CRS onset is approximately 24 hours, patients could be reviewed the morning after infusion, or sooner if the remote system triggers an alert, avoiding a default longer admission. Physical patient attendance could be reduced further with home blood draws, or a move to next-generation skin or sweat patches that measure cytokines such as IFN-γ in real time. Sweat-based SIMOA patches have already tracked TNF-α, IL-10, and IL-6 in ambulatory adults (31), while microneedle “transdermal analysis” patches detected IL-17A and IL-1α in hidradenitis suppurativa (32). These technologies could be combined with simplification of vital sign parameters (such as a move to temperature alone) to further enhance outpatient monitoring capabilities.

A key limitation of our study was low statistical power due to its small sample size. While this did not directly affect the primary outcome — feasibility — it remained a concern for the cytokine analysis and the combined temperature-cytokine model. Further studies with larger cohorts will be key to validate these models and assess generalizability across diverse CAR-T products and patient populations. We believe that our findings, including a direct comparison to standard inpatient care, bolster the case for shifting the site of post–CAR-T care to the outpatient setting, with wearable monitoring. The threshold-based temperature approach provides a simple, immediately deployable method for real-time CRS detection using a single parameter and is recommended for near-term clinical implementation. While optimized temperature thresholds can achieve high sensitivity for CRS detection, such approaches rely on a single physiologic cutoff and do not incorporate molecular biomarkers or temporal biomarker dynamics. Additional wearable-derived variables, including pulse rate, oxygen saturation, respiratory rate, and motion, were collected but were not prioritized in the present analyses; larger cohorts will be needed to determine whether these features provide incremental predictive value beyond temperature alone. The exploratory ML models, which integrate cytokine biomarkers and provide probabilistic risk scores rather than binary alerts, demonstrated potential for complementary CRS prediction but require validation in larger, adequately powered cohorts. In this framework, machine learning approaches are intended not to replace simple fever-based monitoring but to provide a flexible platform for integrating physiologic and molecular signals that may allow earlier or more nuanced CRS risk prediction. A naive majority-class classifier always predicting no-CRS would match or exceed model accuracy, yet detect zero CRS events (0% F1), confirming that our models’ values lie in their ability to identify CRS episodes. Next steps would include the integration of vital signs and cytokines in a larger cohort within a complete outpatient pathway.

## Methods

### Sex as a biological variable.

Our study examined male and female patients, and similar findings are reported for both sexes.

### Study trial design.

Sex and race data were obtained from the electronic medical record and reflect chart-documented clinical demographic information; these classifications were not assigned by the study investigators. Ethnicity data were not consistently available in the source records and, therefore, are not reported.

The primary objective was to evaluate the feasibility of wearable devices for CRS monitoring in patients with RRMM receiving CAR-T, assessed by CRS detection sensitivity and specificity. Secondary objectives were (a) comparing timing of CRS detection with wearable devices to standard care, and (b) device adherence. The exploratory objectives examined CRS prediction accuracy using cytokine and wearable data, and cytokine biomarkers associated with CRS onset.

### Study procedures.

Participants were consented and enrolled upon admission to MSH for CAR-T therapy infusion. After enrollment, they were asked to wear the wearable device continuously from the day of infusion until discharge. Both staff and patients were blinded to the wearable outputs, treating the device as a ‘black box’ that recorded vital signs data alongside standard nursing care (nursing vital signs were recorded and documented in the medical record every four hours).

In addition to wearing their device, participants had blood drawn at predetermined time points: (a) For patients receiving ide-cel: Blood samples were collected pre-infusion, and at 2, 4, 6, and 8 hours post-infusion on Day 1 (D1, the day of infusion), as well as needed (PRN) at the onset of CRS and daily post-infusion until discharge for other correlatives. Capture of at least one time point from the immediate post-infusion hours (2, 4, 6, or 8) was mandatory. (b) For patients receiving non-ide-cel cell therapy products: Blood samples were collected pre-infusion, PRN at the time of CRS, and daily post-infusion until discharge. Because ide-cel–associated CRS typically begins approximately 1 day post-infusion whereas cilta-cel CRS arises a median 7–8 days later, sampling was front-loaded for ide-cel and distributed for cilta-cel; a single uniform schedule was impractical given the product-specific REMS monitoring requirements in place at the time.

### Wearable device and vital sign analysis.

The FDA-approved wearable device (Gen 2, Current Health Inc., Dover, DE) continuously measured pulse rate, oxygen saturation, respiratory rate, motion, and skin temperature from the upper arm, integrated with an axillary temperature patch (Feverscout, VivaLnk Inc, Santa Clara, CA). Sensor data were transmitted to the cloud. Adherence was calculated as the proportion of valid readings over the total length of stay, with emphasis on high-risk CRS periods.

Three approaches for CRS detection using axillary and skin temperature were evaluated: (a) fixed thresholds, (b) individualized thresholds (baseline mean temperature + 2 standard deviations), and (c) a combination of fixed and individualized changes (‘OR’ condition). Baseline temperature was defined as the mean of all temperature values collected by the wearable in the first five hours following therapy administration. Although the wearable also continuously measured pulse rate, oxygen saturation, respiratory rate, and motion, the present analyses focused on temperature because fever is central to the ASTCT definition of Grade 1 CRS and represented the most direct physiologic signal for the primary feasibility endpoint. The limited sample size of this pilot study did not support robust multivariable evaluation of additional wearable-derived predictors.

The analysis focused on detecting the first CRS episode only, to avoid confounding treatment effects on subsequent vital sign changes. Each approach was optimized for sensitivity and specificity, and for a ‘Balanced’ condition, that prioritized sensitivity with a minimum specificity above 0.8. Further details, including ‘balanced’ justification, grid search methods and performance definitions, are in Supplementary Methods.

### Multiplex cytokine assay.

Cytokine profiling was conducted using the Olink proximity extension assay (PEA) platform, a high-throughput multiplex proteomic immunoassay. We used the commercially available Olink Immuno-Oncology panel (Article number 95310), which included 96 immune- and oncology-related proteins (Supplemental Table 4). This technology employes paired oligonucleotide-labeled antibodies that bind bound target proteins, followed by hybridization and amplification via quantitative PCR. The assay outputted protein expression levels as Normalized Protein Expression (NPX) values on a log2 scale.

### Statistical analysis of inflammatory profiles following CAR-T.

To estimate the average daily expression of each inflammatory marker following CAR-T, and compare between groups, a linear mixed-effects regression model for each marker was fitted using the DREAM framework (33). Each model included marker expression as the dependent variable and included time as the independent variable, with the patient’s age as a covariate. Random intercepts across patient ID, and plate ID, were included to account for correlation between repeated measures. Comparisons of interest were performed using contrasts and p-values were adjusted for multiple comparisons using the Benjamini Hochberg approach. The DREAM framework uses an empirical Bayes approach to adjust the t-statistic based on the overall mean and variance of all inflammatory markers, assuming no trend in the data (i.e., not adjusting for systematic changes across markers). Comparisons were deemed as significant at False Discovery Rate (FDR) < 0.05 and Fold-change (FCH) > 1.3, unless stated otherwise.

### Machine learning approach for CRS classification.

The classification outcome was binary CRS status (CRS vs. no-CRS) within a defined prediction window, with the target label derived from clinically documented CRS onset times per ASTCT grading criteria. Data preprocessing was conducted in Python (version 3.8) using pandas and scikit-learn, with forward-fill methods used to address missing data. Cytokine levels were interpolated (linear, spline, and polynomial methods) to match the higher-frequency wearable data. Time-series features were generated by aggregating temperature data into rolling windows of 6 to 14 hours. These longer windows were designed for CRS prediction—forecasting impending CRS by capturing gradual cytokine accumulation and temperature trends—in contrast to the shorter 60-minute windows in the threshold-based analysis, which were optimized for real-time detection of acute temperature elevations. Skin temperature was selected over axillary temperature as the primary input due to its higher sampling rate and ease of future collection.

Five machine learning classifiers (Logistic Regression, Random Forest, Gradient Boosting, Support Vector Machine (SVM), and k-Nearest Neighbor (k-NN)) were evaluated, with hyperparameters tuned using grid search. Combined models incorporating wearable and cytokine data were built using scikit-learn’s Pipeline framework for standardized feature processing. Performance metrics included F1 score (as the primary metric, given class imbalance), accuracy, precision, sensitivity, and specificity, with StratifiedKFold cross-validation (5 splits) to ensure robustness and class weight adjustments to address data imbalances.

SHapley Additive exPlanations (SHAP) were applied to assess feature importance, identifying wearable and cytokine contributions to predictions. Further details on data preprocessing, feature selection, and classifier tuning are provided in the Supplementary Materials.

### Predicting next-day CRS status using daily interferon fold-change.

A statistical classifier was developed to predict the probability of CRS occurring the following day based on a patient’s daily interferon (IFN) fold-change from baseline. Observations included both daily and semi-hourly IFN measurements, depending on the timing of CRS onset. Separate classifiers were trained for cilta-cel (*n* = 16 patients) and ide-cel (*n* = 9), encompassing 49 pre-CRS IFN measurements for ide-cel and 90 pre-CRS measurements for cilta-cel. Missing baseline IFN values were imputed using the mean baseline across all patients.

Two models, logistic regression (linear) and decision tree (non-linear), were trained, with performance evaluated via leave-one-patient-out cross-validation. For each observation, these models estimate the probability that the patient will have CRS before the following observation. Observations with probability exceeding 0.5 were flagged as `predicted to have CRS before next time observation’. Once a prediction was made, subsequent observations for that patient were excluded from the analysis. This approach enabled performance and lead time calculations with 24 hours indicating that CRS was correctly predicted on the day prior to experiencing CRS.

### Study approval.

This investigator-initiated, single-center, single-arm pilot study was conducted at Mount Sinai Hospital (MSH) in New York. The research protocol was reviewed and approved by the Institutional Review Board (IRB) at the Mount Sinai Hospital, New York, USA (Protocol 21-1626). All participants provided written informed consent prior to enrollment. A total of thirty patients were enrolled. Baseline characteristics are summarized in Table 1, and the study schema in Figure 1.

### Data availability.

The deidentified processed wearable-device dataset generated in this study has been deposited in Zenodo under accession 10.5281/zenodo.19740559. The Olink proteomics dataset has been deposited in PRIDE-AP under accession PAD000041. Supporting data values underlying the main figures and Supplemental figures are provided in the Supporting Data Values file.

## Author contributions

S Rajeeve: Concept and design, data acquisition, data analysis, data interpretation, drafting of the manuscript. MW: Data analysis, drafting of the manuscript. NZ: Data analysis. LT: Data analysis. MQ: Data analysis. DP: Data acquisition. NJC: Data analysis. MC: Data analysis. AA handled samples for Olink proteomic analysis. KS: Data acquisition, administrative support. KK: Data acquisition, administrative support. HJ: Data acquisition, administrative support. SA: Data acquisition, administrative support. ST, LS, S Richard, JR, CR, HJC, AC, SJ: Data interpretation, critical review of the manuscript. AL: Concept and design, data interpretation, drafting of the manuscript, supervision, co–corresponding author. ACR: Concept and design, data interpretation, drafting of the manuscript, supervision, co–corresponding author. SP: Concept and design, data interpretation, drafting of the manuscript, supervision, co–corresponding author.

## Conflict of interest

MW, NZ, NJC, and MC were salaried employees of Best Buy Health Inc. and held stock in Best Buy Inc. Boston, Massachusetts (USA) at the time of data collection. MW is presently a salaried employee of Current Health Inc., Boston, Massachusetts (USA). AC receives research support from Janssen and is a consultant and/or advisor for Abbvie; Adaptive; Amgen; Antengene; Bristol Myers Squibb; Forus; Genetech/Roche; Glaxo Smith Klein; Janssen; Karyopharm; Millenium/Takeda; and Sanofi/Genzyme. AR is a consultant for Sanofi, BMS, Janssen and Adaptive. CR is a consultant for Janssen, BMS, Takeda, Sanofi and Artiva. HJC is employed by The Multiple Myeloma Research Foundation and has research support from BMS, Takeda, and Genentech. JR is a consultant/advisor for Janssen, BMS, Pfizer, Karyopharm, Sanofi, Takeda, Abbvie, and is part of speakers bureau for Janssen, BMS, Sanofi, and Adaptive Biotechnologies. SR is also part of BMS Advisory Board/ Steering Committee – Gracell Biotechnologies. SJ is a consultant for Janssen, BMS, Legend Biotech, Regeneron, Caribou, Sanofi, Takeda, and Karyopharm. He is DMC chairman for Genmab and Sanofi. He has membership on an entity’s Board of Directors or advisory committees for IMS, ASH, and SOHO. AL reports research support from Celgene/BMS. SP receives research support from Amgen Inc., Celgene/BMS Corporation, The Multiple Myeloma Research Foundation, GRAIL, and Caribou; he is also on the advisory board for GRAIL and Genentech. SR has received Honoraria from Janssen and research support from Janssen, BMS, C4 Therapeutics, Heidelberg Pharma, and Gracell Biotechnologies.

## Funding support

This work is the result of NIH funding, in part, and is subject to the NIH Public Access Policy. Through acceptance of this federal funding, the NIH has been given a right to make the work publicly available in PubMed Central.

- Bristol Myers Squibb.
- The Multiple Myeloma Center of Excellence Philanthropic Fund.
- ASH Bridge Grant Award (AL).
- National Cancer Institute grants R01 CA244899 and R01 CA252222 (SP).
- Tisch Cancer Institute grant P30 CA196521 (SP).

## Supplementary Material

Supplemental data

ICMJE disclosure forms

Supporting data values

## Figures and Tables

**Table 1:**
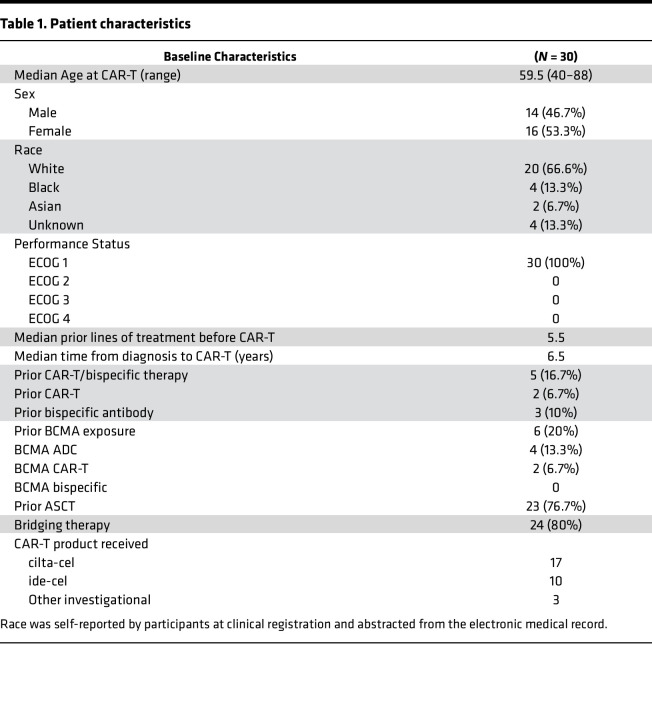
Patient characteristics

**Table 2:**
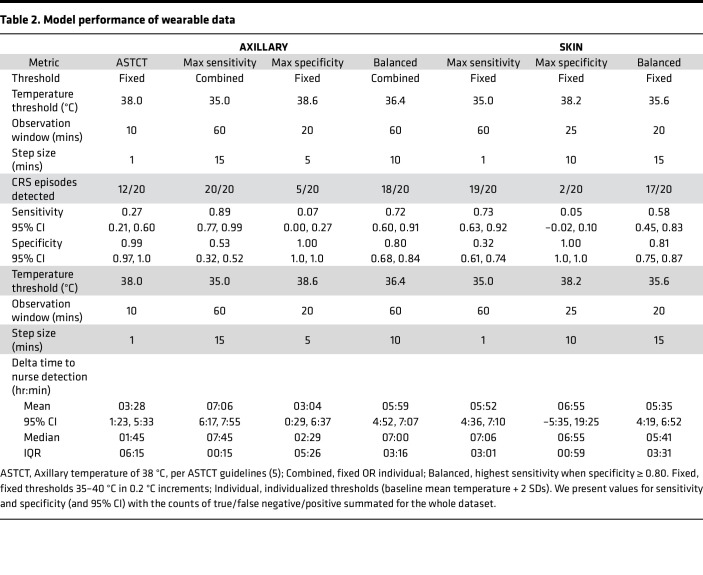
Model performance of wearable data

**Table 3:**
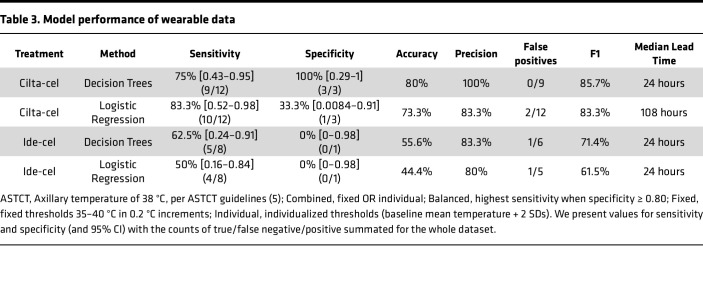
Model performance of wearable data

## Version 1 05/05/2026

In-Press Preview

## Version 2 06/22/2026

Electronic publication
