# Supplementary material for: Detection of cytokine release syndrome using wearables and cytokine profiling following CAR-T therapy for myeloma
Source: JCI Insight. 2026 May 5;11(12):e203988. doi: 10.1172/jci.insight.203988 (PMC13313484; doi:10.1172/jci.insight.203988)
Supplement: Supplemental data [file jciinsight-11-203988-s190.pdf]

EARLY DETECTION OF CYTOKINE RELEASE SYNDROME USING WEARABLE DEVICES AND CYTOKINE PROFILING FOLLOWING CAR-T THERAPY FOR MYELOMA

Sridevi Rajeeve[1], Matt Wilkes[2], Nicole Zahradka[2], Lewis Tomalin[3], Mujahid Quidwai[4], Darren Pan[5], Nicholas J Calafat[2], Martin Cusack[2], Adolfo Aleman[6,7], Kseniya Serebryakova[6,7], Katerina Kappes[6,7], Hayley Jackson[6,7], Sarita Agte[8], Santiago Thibaud[6,7], Larysa Sanchez[6,7], Shambavi Richard[6,7], Joshua Richter[6,7], Cesar Rodriguez[6,7], Hearn Jay Cho[6,7], Ajai Chari[5], Sundar Jagannath[6,7], Alessandro Laganà[4,7,9,*], Adriana C Rossi[6,7,*], Samir Parekh[6,7,*].

[1]Myeloma & Cellular Therapy Services, Memorial Sloan Kettering Cancer Center, New York, NY

[2]Best Buy Health, Inc., Boston, MA

[3]Department of Population Health Science and Policy, Icahn School of Medicine at Mount Sinai, New York, NY

[4]Department of Oncological Sciences, Icahn School of Medicine at Mount Sinai, New York, NY

[5]University of California San Francisco, San Francisco, CA

[6]Department of Medicine, Hematology and Medical Oncology, Icahn School of Medicine at Mount Sinai, New York, NY

[7]Tisch Cancer Institute, Icahn School of Medicine at Mount Sinai, New York, NY

[8]Dorset County Hospital, NHS Foundation Trust, Dorchester, United Kingdom

[9]Department of Genetics and Genomic Sciences, Icahn School of Medicine at Mount Sinai, New York, NY

* Corresponding authors

SUPPLEMENTARY METHODS

WEARABLE DEVICE AND VITAL SIGNS ANALYSIS

The FDA 510(k) cleared wearable device (Current Health Gen 2, Best Buy Health Inc., Boston) continuously measured pulse rate, oxygen saturation, covered skin temperature, motion, and respiratory rate from the participants' upper arms. The system also recorded peripheral temperature using an axillary temperature patch (Feverscout, Vivalnk, Campbell, USA), which took readings at a maximum rate of every 2 minutes. The wearables transmitted sensor data via a 'home hub,' using either Wi-Fi or roaming cellular connections to upload data to the cloud. The raw sensor data from the upper arm wearable were processed into vital signs, outputting pulse rate, oxygen saturation, covered skin temperature, and motion observations every two seconds, and respiratory rate every four seconds.

Adherence was defined as the number of observations recorded by the wearable during the participant's stay, divided by the total number of possible observations. Adherence was calculated overall, and during the highest-risk periods for cytokine release syndrome (CRS) based on product. For patients receiving ide-cel or investigational products, high-risk periods were defined as days 0-5 post-infusion, and for cilta-cel, days 5-11.

Three approaches were evaluated for detecting CRS using axillary and skin temperature data recorded by the wearable:

1. Fixed thresholds ranging from 35-40°C in 0.2°C increments.
2. Individualized thresholds based on baseline temperature + 2 standard deviations (SD).
3. A combined approach using both fixed and individualized thresholds (an "OR" condition).

Baseline temperature was defined as the mean of all temperature values collected in the first five hours following therapy administration.

A grid search was conducted to calculate sensitivity, specificity, and time to nurse detection for each threshold approach, incorporating two additional variables: *observation window* and *step size*. The observation window was the time-period over which the axillary and skin temperatures were aggregated (5, 15, 30, 45, and 60 minutes), and step size was the interval between consecutive observation windows (1, 5, 10, and 15 minutes) (**Supp Figure S2**).

We assessed performance (threshold breach vs. CRS) at each step, using the following definitions:

- True positive: Any threshold breach occurring < 8 hours before nurse detection.
- False negative: No threshold breach < 8 hours before nurse detection.
- False positive: Any threshold breach > 8 hours before nurse detection (in patients who developed CRS); any threshold breach during the patient's stay (in patients who did not develop CRS).
- True negative: No threshold breach > 8 hours before nurse detection or the first true positive (in patients who developed CRS); no threshold breach during the patient's stay (in patients who did not develop CRS).

Multiple breaches within an hour of each other were combined into a single event, reflecting the clinical use of a monitoring-based alarm system, where immediately repeated alerts would not result in additional awareness of the event. After a true positive occurred, other observations between nurse detection minus eight hours and the time of the true positive were no longer assessed. Eight hours was chosen as equivalent to two rounds of nursing observations in standard care. We note that our approach was conservative, as we were evaluating our performance more often than nurses, so were penalized more heavily for false events.

Performance was evaluated based on sensitivity, specificity, CRS event detection, and time between model detection and standard nursing care. While there are no universal criteria for sensitivity and specificity in remote patient monitoring alerts, we prespecified a specificity of >0.8 for model selection. Specificity is particularly important in home settings compared to hospitals, as false alarms at home could unnecessarily disrupt patients or caregivers, lead to unwarranted hospital visits, or result in paramedic dispatch[1]. If sensitivity or specificity is pushed too far in either direction, it can lead to a loss of trust from patients and clinicians, financial burdens, and ultimately a decline in the quality of care. We selected 0.8 as the minimum specificity required to increase a pre-test probability from "uncertain" to "likely" using a categorical application of Bayes' theorem[2,3]. The analysis focused on detecting the first episode of CRS only. This was done due to the lack of a clear definition of when one episode ended and another began, as well as to avoid the confounding effects of CRS treatment on subsequent vital sign changes.

MACHINE LEARNING APPROACH FOR CRS CLASSIFICATION

To enhance CRS onset prediction in patients receiving CAR-T therapies, we designed and implemented a systematic machine learning approach integrating both cytokine biomarkers and wearable device data. Our methodology encompassed four distinct models targeting two CAR-T products: (1) ide-cel Model with Cytokines Only, (2) cilta-cel

Model with Cytokines Only, (3) ide-cel Model with Cytokines and Wearable Data, and (4) cilta-cel Model with Cytokines and Wearable Data.

Data Collection and Preprocessing

Patient data were collected through two primary streams: standard laboratory cytokine assays and continuous wearable device monitoring. Cytokine measurements were obtained pre- and post-infusion for both ide-cel and cilta-cel CAR-T products. Wearable metrics included continuous skin temperature and axillary temperature readings. We prioritized skin temperature data as our primary physiological marker due to its superior temporal resolution, data completeness and ease of future collection compared to axillary temperature. Data preprocessing, implemented using Python 3.8 with pandas (version 2.2.3) and scikit-learn (version 1.5), included missing data imputation using forward-fill methods, feature standardization using StandardScaler, and time-series alignment between cytokine and temperature data.

Feature Engineering and Data Integration

To address the temporal resolution mismatch between daily cytokine measurements and minute-level temperature readings, we implemented a comprehensive interpolation strategy using pandas. We evaluated linear, spline, and polynomial interpolation methods, validating interpolated values against known measurements. Time-series features were engineered using rolling windows (numpy.roll) across multiple intervals (6, 8, 10, 12, and 14 hours). The selection of skin temperature as our primary wearable feature was based on quantitative analysis including signal-to-noise ratio assessment, missing data impact evaluation, temporal coverage analysis, and cross-correlation with clinical outcomes. Data integration was achieved using pandas.merge with time-based joining, implementing careful handling of missing values through forward-fill methods. Additionally, time-lagged features were engineered by computing the rate of change between consecutive measurements at multiple lag intervals: for each cytokine C at time t, $\Delta C = C(t) - C(t-lag)$ for lag $\in$ {6h, 12h, 24h}. These features captured dynamic biomarker trajectories rather than static levels alone.

Model Implementation and Classifier Selection

We evaluated five machine learning classifiers implemented through scikit-learn: Logistic Regression (C=1.0, max_iter=1000, class_weight='balanced'), Random Forest (n_estimators=100, max_depth=None), Gradient Boosting (learning_rate=0.1, n_estimators=100), Support Vector Machines (kernel='rbf', C=1.0), and k-Nearest Neighbors (n_neighbors=5, weights='uniform'). For cytokine-only models, Gradient Boosting emerged as the optimal classifier for ide-cel, achieving 76.47% accuracy (precision=86%, sensitivity=77%, specificity=67%), while Random Forest performed best for cilta-cel with 78.57% accuracy (precision=86%, sensitivity=78%, specificity=70%).

Performance Metrics and Model Validation

Model performance was evaluated using a comprehensive set of metrics calculated through sklearn.metrics. These included accuracy_score for overall accuracy, precision_recall_curve for precision and sensitivity assessment, and confusion_matrix for specificity calculation (implemented as TN/(TN+FP)). Cross-validation was performed using StratifiedKFold with 5 splits to ensure robust performance estimation. The combined models, integrating both cytokine and temperature data, were implemented using scikit-learn's Pipeline framework, incorporating StandardScaler and the respective optimal classifiers for each CAR-T product.

Feature Importance and Model Interpretation

Feature importance analysis was conducted using SHAP (SHapley Additive exPlanations, version 0.46) with TreeExplainer for tree-based models and KernelExplainer for other algorithms. SHAP values were aggregated across cross-validation folds to ensure stability in feature importance rankings. For the ide-cel model, key predictive markers included IL5, IFN-gamma, NOS3, CD4, and TNFRSF9, while cilta-cel's significant features included IL10, CCL4, MCP-2, MCP-3, and IFN-gamma. Temperature, as a time-series feature, demonstrated consistent importance across both models.

Model Performance and Limitations

The integration of wearable temperature data with cytokine measurements showed mixed effects on prediction performance within the 6-hour window: while overall accuracy increased, precision and sensitivity declined, indicating that temperature data may help reduce false positives at the cost of reduced true-positive detection. The ide-cel combined model achieved 84.62% accuracy (precision=66.67%, sensitivity=50%, specificity=66.67%), while the cilta-cel combined model reached 80.62% accuracy (precision=43%, sensitivity=38%, specificity=88%). To address dataset imbalance challenges, we implemented class-weighted models and evaluated predictive stability across multiple cross-validation folds. The interpolation of cytokine measurements to match the higher frequency of temperature data enabled dynamic tracking of CRS risk, though this approach necessitates further validation in larger patient cohorts.

SUPPLEMENTARY FIGURES

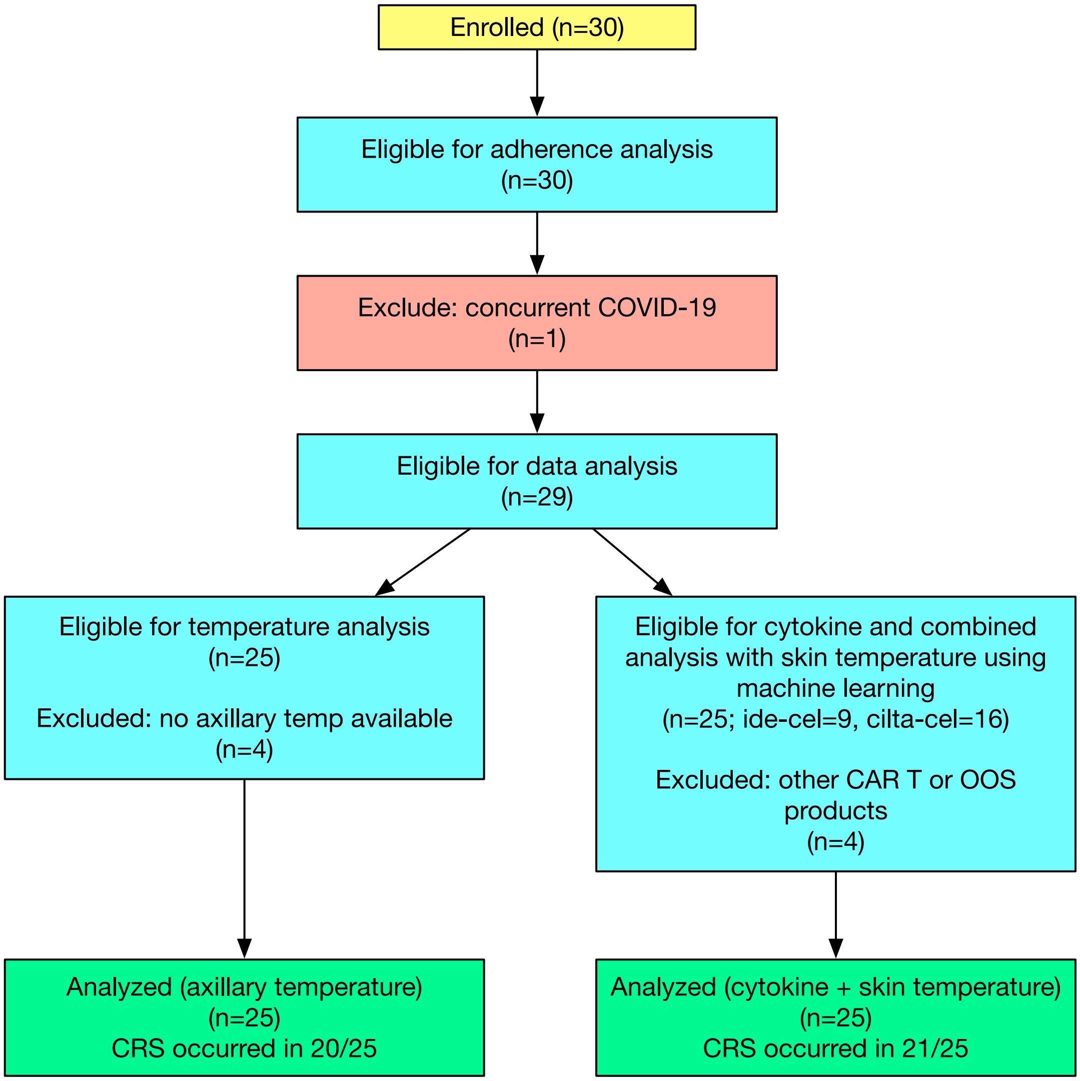

Fig. S1. CONSORT diagram showing the flow of participants through each stage of the study. The OOS (out-of-specification) CAR T cases were excluded from the analysis because they did not meet the predefined quality standards required for clinical use, such as cell viability, purity, or concentration, as set by regulatory guidelines. Including these cases could have introduced variability and potential biases, as they do not represent the standard treatment administered to patients. Our goal was to ensure the analysis reflected the outcomes of CAR T therapies that met regulatory specifications and were deemed safe and effective for clinical application.

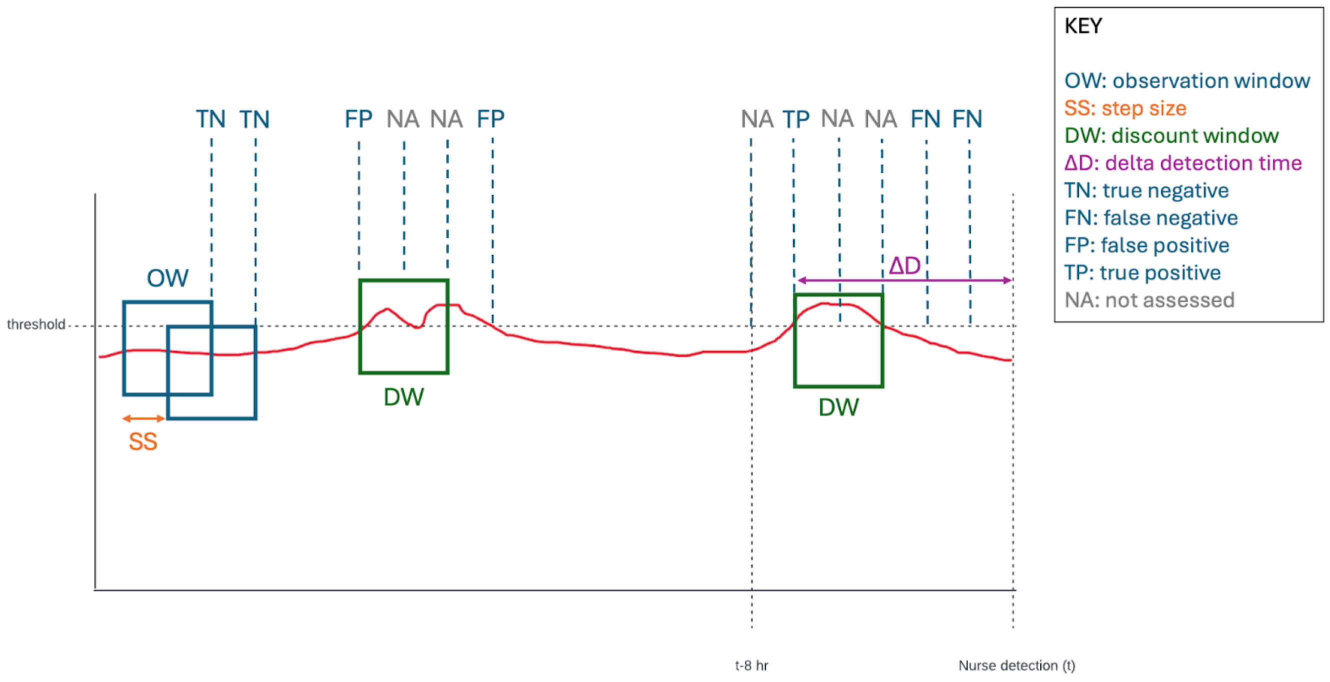

Fig. S2. Schematic depiction of the measured intervals and the approach to CRS detection classification in the wearable analysis. The red line depicts temperature, the ‘threshold’ is the temperature at which a positive, either true or false, was assigned at each point of evaluation.

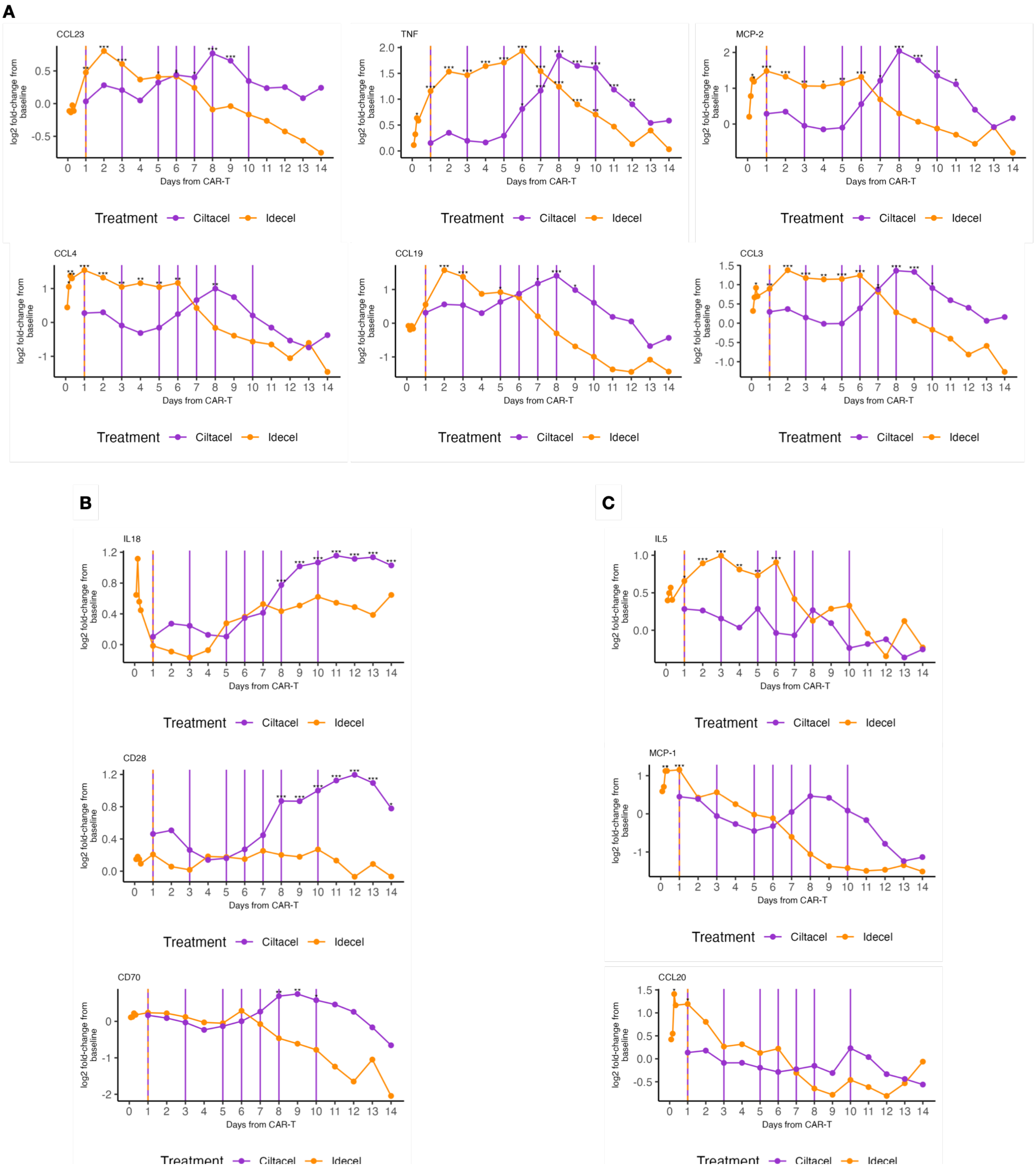

Fig. S3. Olink analysis revealed cytokines significantly induced by specific treatments. (A-C) Plots show expression of example inflammatory markers inflammatory following cilta-cel and ide-cel. **(A)** Shows markers that change following both treatments, **(B)** shows markers that are specific to cilta-cel, and **(C)** shows markers specific to ide-cel.

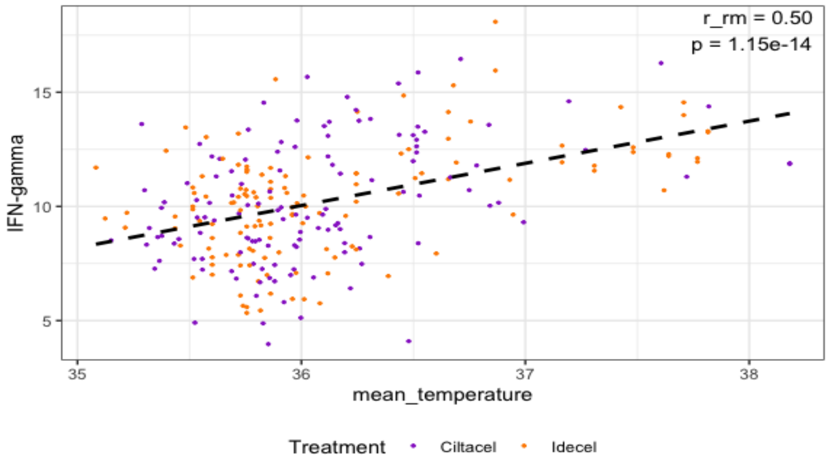

Fig. S4. Relationship between IFN-γ levels and body temperature measured by wearable devices. Scatter plot showing daily IFN-γ levels (NPX units, Olink platform) versus mean daily axillary temperature recorded by wearable devices. Each point represents a daily observation and is colored by CAR-T product (cilta-cel or ide-cel). The dashed line indicates the repeated-measures correlation fit accounting for within-patient observations ($r = 0.50$, $p = 1.15 \times 10^{-14}$).

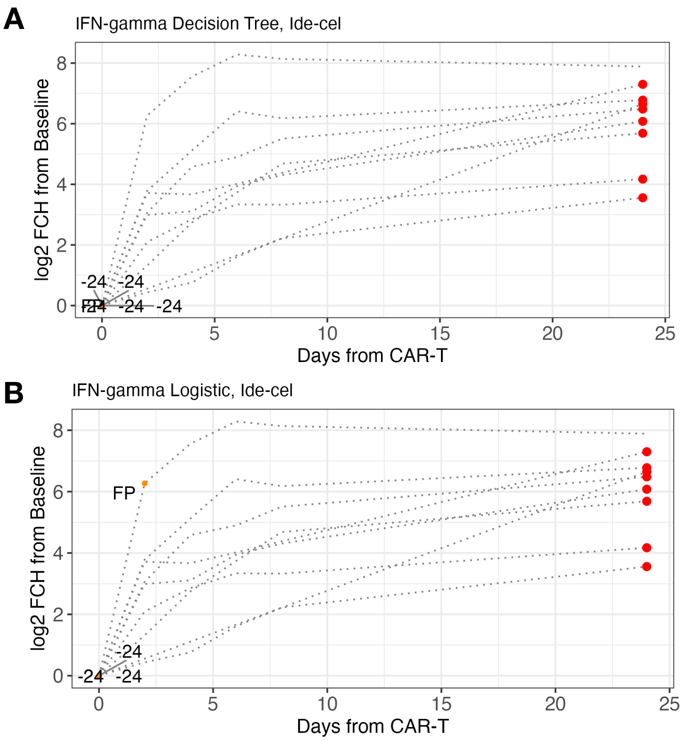

Fig. S5. IFN-γ Fold-Change as a Predictive Biomarker for CRS in ide-cel. Decision tree model **(A)** and logistic regression model **(B)** using IFN-γ $\log_2$ fold-change from baseline in patients treated with ide-cel. Points represent individual observations, and dashed curves illustrate the fitted decision boundaries of the model across time from CAR-T infusion. The red points indicate the day that CRS occurred for each patient, orange labels indicate lead time for each patient, 'FP' indicates False Positive (i.e. patient never experienced CRS during the study).

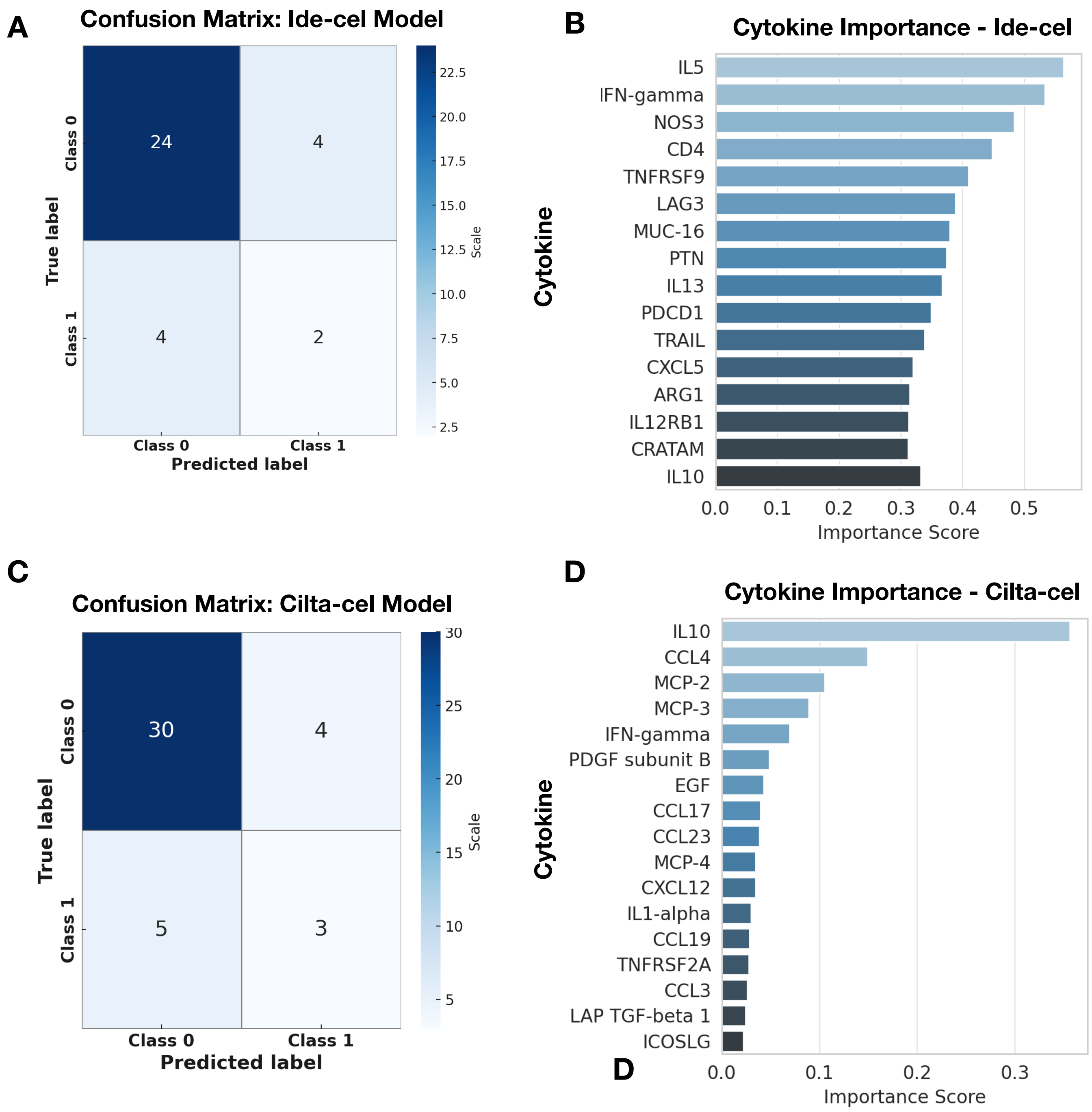

Fig. S6. Performance and key cytokines in ML analysis. (A,C) Confusion matrices for ide-cel and cilta-cel cytokine models. **(B,D)** Top cytokines for ide-cel and cilta-cel ranked by importance as calculated using the sckitlearn package.

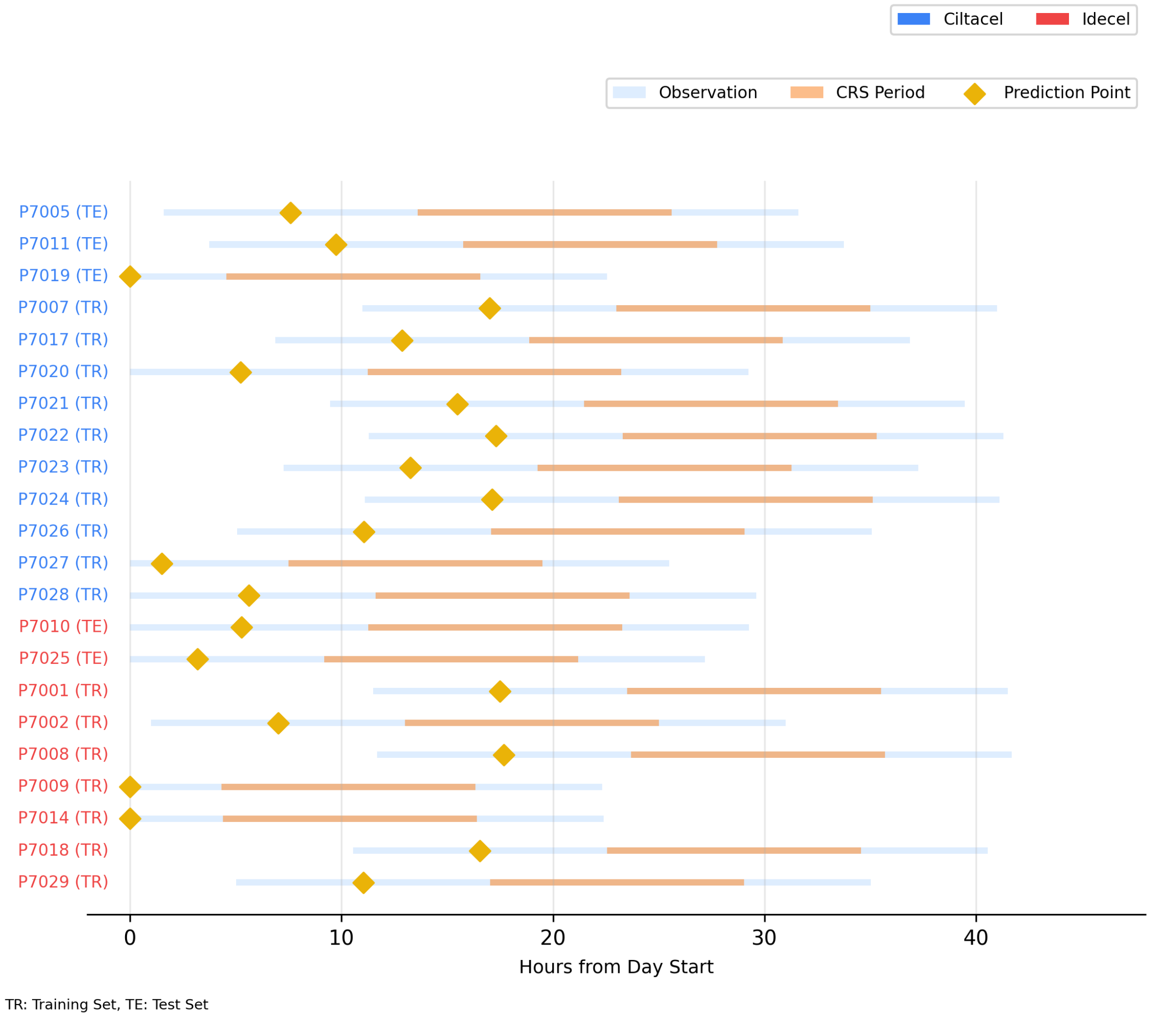

Fig. S7. Machine learning approach to predict CRS. Swimmers plot for combined skin temperature + cytokine machine learning model.

SUPPLEMENTARY TABLES

Table S1. Patient characteristics.

Patient_ID	Age	Sex	CAR T product	CRS (0 = No, 1= Yes)	CRS grade	Included in Temp Analysis	Included in Cytokine and ML analysis
WEAR_7001	83	F	ide-cel	1	1	Y	Y
WEAR_7002	68	M	ide-cel	1	1	Y	Y
WEAR_7003	67	M	Other investigational	1	1	Y	N
WEAR_7004	59	M	cilta-cel	0	NA	Y	Y
WEAR_7005	59	F	cilta-cel OOS	1	1	Y	N
WEAR_7006	48	M	ide-cel	0	NA	Y	Y
WEAR_7007	65	M	cilta-cel	1	1	Y	Y
WEAR_7008	68	F	ide-cel OOS	1	1	Y	N
WEAR_7009	75	M	ide-cel	1	3	Y	Y
WEAR_7010	50	F	ide-cel	1	1	Y	Y
WEAR_7011	51	M	cilta-cel	1	1	N	Y
WEAR_7012	66	M	cilta-cel	0	NA	Y	Y
WEAR_7013*	67	F	Other investigational	1	1	N	N
WEAR_7014	73	M	ide-cel	1	1	Y	Y
WEAR_7015	58	M	Other investigational	0	NA	Y	N
WEAR_7016	78	F	cilta-cel	0	NA	Y	Y
WEAR_7017	65	M	cilta-cel	1	1	Y	Y
WEAR_7018	82	M	ide-cel	1	1	Y	Y
WEAR_7019	67	F	cilta-cel	1	2	Y	Y
WEAR_7020	60	F	cilta-cel	1	4	Y	Y
WEAR_7021	54	F	cilta-cel	1	1	Y	Y
WEAR_7022	40	F	cilta-cel	1	1	Y	Y
WEAR_7023	55	F	cilta-cel	1	1	Y	Y
WEAR_7024	60	M	cilta-cel	1	1	N	Y
WEAR_7025	50	F	ide-cel	1	1	Y	Y
WEAR_7026	62	M	cilta-cel	1	NA	N	Y
WEAR_7027	62	M	cilta-cel	1	1	Y	Y
WEAR_7028	74	F	cilta-cel	1	1	Y	Y
WEAR_7029	88	M	ide-cel	1	1	Y	Y
WEAR_7030	53	F	cilta-cel	1	1	N	Y

*WEAR_7013 was excluded for concurrent COVID-19.

Table S2. Best performing balanced model.

Metric	Overall	Ide-cel	Cilta-cel	Other experimental products
**Total patients**	50	10	17	3
**Patients included in the temperature analysis**	25	10	13	2
**Patients who developed CRS**	20	9	10	1
**CRS episodes detected**	18	9	8	1
**Sensitivity**	0.72	0.91	0.55	1
**Specificity**	0.8	0.73	0.82	0.74
**Delta time to nurse detection** Mean, Median [IQR], (hr:min)	5:59, 7:00 [3:16]	5:54, 7:21 [4:30]	6:08, 7:15 [2:57]	5:40, 5:40 [0]

Axillary temperature "Combined": fixed (36.4 C) or individual threshold, observation window 60 mins, step size 10 mins) in the 25 patients included in the analysis; 'ide-cel': idecabtagene vicleucel; 'cilta-cel': ciltacabtagene autoleucel; 'Other experimental products': GPRC5D CAR-T, Caribou AlloCAR-T, Next-Gen CAR-T.

Table S3. Performance metrics for CRS classification models.

F1 score is reported as the primary metric given class imbalance in the observation-level data (see main text). The majority class (no-CRS) baseline is included for comparison. Observation counts for multi-cytokine models were derived from confusion matrices (Supp. Fig. S4); for IFN-γ models, from pre-CRS measurement counts reported in Methods. F1 = 2×Precision×Sensitivity / (Precision + Sensitivity). N/A = not applicable.

Panel A: Multi-cytokine ML models (cytokine-only vs. cytokine + temperature)

Model	BestClassifier	Npts	Nobs	F1	Acc	Prec	Sens	Spec	Prediction Window
Ide-celCytokines only	Gradient Boosting	9	34	**81%**	77%	86%	77%	67%	N/A
Cilta-celCytokines only	Random Forest	16	42	**82%**	79%	86%	78%	70%	N/A
Ide-celCyto + Temp	Random Forest	9	34	**57%**	85%	67%	50%	67%	6 hours
Cilta-celCyto + Temp	Gradient Boosting	16	42	**40%**	81%	43%	38%	88%	6 hours
Ide-celMajority classbaseline	—	9	34	**0%**	82%	N/A	0%	100%	—
Cilta-celMajority classbaseline	—	16	42	**0%**	81%	N/A	0%	100%	—

Majority class baseline: a naïve classifier that always predicts no-CRS (the majority class at the observation level, comprising 82% of ide-cel and 81% of cilta-cel observations). Observation counts derived from confusion matrices in Supp. Fig. S4 (ide-cel: 24+4+4+2 = 34; cilta-cel: 30+4+5+3 = 42).

Panel B: IFN-γ fold-change classifiers (single-biomarker models, from Figure 3C)

Product	Classifier	Npts	Nobs	F1	Acc	Prec	Sens	Spec	FP	Median Lead Time
Cilta-cel	Decision Tree	16	90	**85.7%**	80%	100%	75%(9/12)	100%(3/3)	0/9	24 hours
Cilta-cel	Logistic Regression	16	90	**83.3%**	73.3%	83.3%	83.3%(10/12)	33.3%(1/3)	2/12	108 hours
Ide-cel	Decision Tree	9	49	**71.4%**	55.6%	83.3%	62.5%(5/8)	0%(0/1)	1/6	24 hours
Ide-cel	Logistic Regression	9	49	**61.5%**	44.4%	80%	50%(4/8)	0%(0/1)	1/5	24 hours

Acc = accuracy; Prec = precision; Sens = sensitivity; Spec = specificity; FP = false positives. The ide-cel IFN-γ models show 0% specificity because only 1 non-CRS patient was in the ide-cel cohort. Observation counts reflect pre-CRS IFN-γ measurements (49 for ide-cel, 90 for cilta-cel) as reported in Methods. Performance evaluated via leave-one-patient-out cross-validation.

Table S4. Olink immuno-oncology cytokine panel.

Olink Immuno-Oncology panel				
IL8	CXCL9	PDGF subunit B	IL10	ICOSLG
TNFRSF9	CD8A	PDCD1	TNFRSF12A	MMP12
TIE2	CAIX	FASLG	CCL23	CXCL13
MCP-3	MUC-16	CD28	CD5	PD-L2
CD40-L	ADA	CCL19	CCL3	VEGFA
IL-1 alpha	CD4	MCP-2	MMP7	IL4
CD244	NOS3	CCL4	ARG1	LAG3
EGF	IL2	IL15	NCR1	IL12RB1
ANGPT1	Gal-9	Gal-1	DCN	IL13
IL7	VEGFR-2	PD-L1	TNFRSF21	CCL20
PGF	CD40	CD27	TNFRSF4	TNF
IL6	IL18	CXCL5	MIC-A/B	KLRD1
ADGRG1	GZMH	IL5	CCL17	GZMB
MCP-1	KIR3DL1	HGF	ANGPT2	CD83
CRTAM	LAP TGF-beta-1	GZMA	PTN	IL12
CXCL11	CXCL1	HO-1	CXCL12	CSF-1
MCP-4	TNFSF14	CX3CL1	IFN-gamma	
TRAIL	IL33	CXCL10	LAMP3	
FGF2	TWEAK	CD70	CASP-8	

REFERENCES

1. Wilkes, M. *et al.* Hospital is not the home: Lessons from implementing remote technology to support acute inpatient and transitional care in the home in the United States and United Kingdom. *J. Med. Internet Res.* **26**, e58888 (2024).
2. Medow, M. A. & Lucey, C. R. A qualitative approach to Bayes' theorem. *Evid. Based. Med.* **16**, 163–167 (2011).
3. Power, M., Fell, G. & Wright, M. Principles for high-quality, high-value testing. *Evid. Based. Med.* **18**, 5–10 (2013).
